# Antifungal activity of lactobacilli isolated from Armenian dairy products: an effective strain and its probable nature

**DOI:** 10.1186/s13568-018-0619-y

**Published:** 2018-05-28

**Authors:** Inga Bazukyan, Lusine Matevosyan, Anna Toplaghaltsyan, Armen Trchounian

**Affiliations:** 1 Department of Biochemistry, Microbiology & Biotechnology, Faculty of Biology, Yerevan State University, 1 Alex Manoogian Str., 0025 Yerevan, Armenia; 2 Present Address: Laboratory of Strain-Producers and Biosynthesis of Biologically Active Materials, Scientific and Production Center “Armbiotechnology”, National Academy of Sciences of Armenia, 14 Gyurjyan Str., 0056 Yerevan, Armenia

**Keywords:** Lactobacilli, Antifungal activity, External factors, Cell wall, Antifungal protein nature

## Abstract

Different strains of lactobacilli isolated from Armenian dairy products (matsoun, sour cream and different types of cheeses), were studied for antifungal activity. *Lactobacillus rhamnosus* MDC 9661 strain was shown to have inhibitory activity against *Penicillium aurantioviolaceum* and *Mucor plumbeus* growth. Bacterial cell-free supernatant didn’t show antifungal activity. The *L. rhamnosus* antifungal activity was stable to the wide range of pH from 3 to 10. This activity was high after treatment with both low temperature (− 30 °C) and lysozyme and with ultrasound. However, it was sensitive to high temperature from 45 to 80 °C and proteolytic enzymes. The results suggest the proteinaceous nature of *L. rhamnosus* antifungal activity associated with bacterial cell wall. *L. rhamnosus* MDC 9661 could be recommended as a starter for production of dairy products, functional food and preserving strain in food production.

## Introduction

Food and feed spoiling moulds and yeasts can be potential hazards and cause great economic losses worldwide (De Muynck et al. 2004). The reduction of mould and yeast growth in food and feed production and their storage is of the primary importance. There is a great interest in developing efficient and safe strategies for this purpose. Thus, the application of biological preservation has received much attention in recent years. The natural origin and the good examination of producers which were isolated from nature are the primary demands to these new agents. The best candidates satisfying these demands are lactic acid bacteria (LAB) (Gerez et al. 2009).

The great variety of ecological-geographic conditions of Armenia with its sharply defined vertical zoning promotes the development of unique associations of LAB in traditional dairy products. During centuries Armenians have prepared traditional protein-enriched dairy products matsoun, sour cream and different types of cheeses, having significant physiological, antagonistic, antioxidant and antiallergenic activity (Bazukyan et al. 2010; Movsesyan et al. 2010). Inside gastrointestinal tract LAB suppress unwanted microbiota growth due to production of various antimicrobial substances and ability to lower the environment’s pH.

There are many substances synthesized by LAB with antibacterial activity such as hydrogen peroxide, carbon dioxide, diacetyl, bacteriocins and proteolytic enzymes (Gourama 1997; Guo et al. 2011; Laref and Guessas 2013). The antagonistic properties of matsoun are usually explained by its acidic pH, because of synthesis of carboxilic (lactic acid particularly) and fatty acids (Dalie et al. 2010; Falguni et al. 2010). The latter’s are known as the most common antifungal components. Antibacterial activity of LAB has been established well (Mauch et al. 2010), but LAB inhibition of yeasts and moulds growth has yet to be studied (De Muynck et al. 2004; Laref and Guessas 2013). Antifungal activity of LAB is low and the nature of such activity is still unclear.

In the present study, we have investigated the antifungal activity of lactobacilli strain isolated from matsoun and conducted preliminary studies on the nature of these substances.

## Materials and methods

### Objects of investigation

The objects of investigation were different LAB strains isolated from dairy products: *Lactobacillus rhamnosus* R-2002 (the accession number is KY054594 submitted in GenBank), *L. delbrueckii* subsp. *lactis* INRA-2010-4.2 and *L. delbrueckii* subsp. *bulgaricus* INRA-2010-5.2 stored at Microbial Depository Center (MDC) (Scientific and Production Center “Armbiotechnology”, National Academy of Sciences of Armenia, Yerevan, Armenia, WDCM 803), under the numbers MDC 9661, MDC 9632 and MDC 9633, respectively, the strains RIN-2003-Ls and BAM-2003-Lb were identified as *Lactobacillus delbrueckii* subsp. *bulgaricus*, the strains INA-5.1 and INA 21.1 were identified as *Lactobacillus rhamnosus*, and INRA-2010-H11 as *Lactobacillus helveticus* according to multiphase approach comprising classical and molecular methods (Bazukyan 2018). All strains were re-cultivated in skim milk medium. The skim milk medium was reconstituted (10% w/v) from milk powder (Katnarat, Armenia) and then sterilized at 110 °C for 15 min. The stock cultures of LAB were kept at − 20 °C in De Man, Rogosa and Sharpe (MRS, Hi-Media, India) broth containing 20% w/v glycerol. Before use, the strains were propagated twice in the appropriate broth and cultivated overnight at optimal temperature (30–42 °C).

For study of antifungal activity all isolated strains were cultivated in modified MRS media with followed composition: peptone 10 g/L, meat extract 10 g/L, yeast extract 5 g/L, glucose 20 g/L, ammonium citrate 2 g/L, MgSO_4_ 0.05 g/L, Tween-80 0.8% v/v, agar–agar 1.5% w/v. All reagents used were produced by Hi-Media, India.

#### Study of antifungal activity and its dependence on external factors

Antifungal properties of the studied were determined by well-diffusion assay and total diffusion into agar. All experiments had been done with the modified MRS media because it was efficient for the growth of fungi and LAB. At the same time, there are no particular media suitable for the cultivation of fungi, (e.g. Saburo media) on which one can obtain relevant growth of LAB. Various groups of fungi were used as test-organisms: *Candida guilliermondii* NP 4 (provided by Prof. L. Navasardyan, Department of Biochemistry, Microbiology and Biotechnology, Yerevan State University, Yerevan, Armenia) (Navasardyan et al. 2017), *Candida albicans* 301 (isolated from clinical material), *Debaryomyces hansenii*, *Mucor plumbeus*, *Geotrichum candidum, Fusarium* sp. CB1853, *Cladosporium* sp.(isolated from spoiled food) [identified and provided by Biopolymers Interaction Assemble, Function and Interaction of Proteins Laboratory (FIRL), National Research Institute of Agronomy (INRA), Nantes, France] (Ahmadova et al. 2013), *Aspergillus flavus, Penicillium aurantioviolaceum* and *Trichoderma viride* (isolated from spoiled food and identified by Dr. K. Grigoryan, Research Institute of Biology, Yerevan State University, Yerevan, Armenia).

Using well-diffusion method (Ndagano et al. 2011) the suspension with fungal spores (10^4^ spores per mL) or 0.1 mL of yeasts cultural liquid (which contained 10^4^ cells per mL) was added in Petri dish and covered by modified MRS 1.5% w/v of agar. Then, LAB were grown for 24 h in MRS medium and 0.1 mL of each LAB cultural liquid was added to wells both in native conditions and after adjustment of pH to 6.5. Antifungal effect of bacterial cell-free supernatant was determined by the same method. The antifungal activity was determined by the absence of fungal/yeasts growth around well. Using diffusion into agar, 70 μL overnight LAB culture was added to small (7 mL) Petri dishes and covered by modified MRS agar. Over 48 h, suspensions of fungal spores were dropped on the nutrient medium surface. The suspensions of fungal spores were prepared after 7–10 days of fungi growth, until full sporulation, after which the spores were collected by plastic microbiological loop in 100 mM phosphate buffer (pH 6.5), and the quantity of spores was determined in counting chamber “Malassez”(Germany). The quantity of spores was adjusted to 10^4^ per mL.

The effect of cultivation duration on antifungal activity was established by growing LAB during 24 and 48 h on modified MRS medium. For testing pH stability, after cultivation during 48 h the pH of LAB cultural media was adjusted to different pH values (from 3 to 10, by concentrated HCl or 40% w/v NaOH), and then the antifungal activity was determined. For testing the temperature stability, after cultivation during 48 h the cultural liquids of LAB were treated at different temperatures in the range of 45–80 °C during 15 min, and then the antifungal activity was determined.

The antifungal activity of concentrated (10×) supernatant by evaporation in “Universal Vacuum System UVS 800 DDA Speed Vac GMI” (USA), as well as antifungal activity of concentrated (22.5×) supernatant by lyophilization in “HETO DRY WINNER DW 6-85-1” (Serial N 21057B, Denmark) at − 90 °C and at 6–7 mbar vacuum was determined by the well diffusion method.

#### Determination of antifungal activity linked with bacterial cell wall

The antifungal activity linked with bacterial cell wall was determined after 48 h of cultivation, when LAB biomass was separated from cultural liquid and washed twice with 0.9% NaCl. Then biomass was treated with 10 mg/mL lysozyme and 70 MHz ultrasound using sonificator (“Labsonic 2000, B. Braun”, Germany). The cell walls were separated from cytoplasm content by centrifugation. After separation, approximately 0.1 mL of cell walls were added to sterile Petri dishes and covered with modified MRS agar. After polymerization the 10^4^ moulds spores were added on the surface of media.

Two controls were used. The first was the growth of 10^4^ fungi spores on the surface of modified MRS. The second was the growth of fungi spores on the surface of modified MRS with addition of spores and cells free supernatant of fungi and 1% of LAB. Spores and cell-free supernatant was obtained by filtration of cultural liquid through 0.2 nm filter. The spores and cell-free supernatant of all filamentous fungi, even if antifungal activity had not been established against them, were used as elicitors.

#### Determination of the nature of the antifungal activity and purification of its component

LAB antifungal activity nature was evaluated by the samples treatment with different enzymes. The effect of enzymes was determined by the incubation of cell suspension with proteinase K and trypsin at final concentration of 0.1 mg/mL in 20 mM phosphate buffer (pH 7.0), and pepsin at 0.1 mg/mL in 20 mM glycine–HCl (pH 2.0). After the incubation for 24 h the reactions were stopped by centrifugation.

The partial purification of antifungal component was made only from LAB cells, but not from supernatant. After 48 h of LAB cultivation in the modified MRS broth the biomass was separated by centrifugation during 5 min at 11,200*g*. The biomass was washed twice with 0.9% NaCl and resuspended in distilled water. The suspension was treated by freezing at − 20 °C overnight. Afterwards, the tubes were incubated at 63 °C for 30 min. Then biomass was treated by 10 mg/mL lysozyme during 1 h at 37 °C and 70 MHz ultrasound “Labsonic 2000, B. Braun” (Germany). The cell walls were separated from cytoplasm content by centrifugation at 15,000*g* during 5 min. After separation, both supernatant and cell walls were treated by addition of 4 M urea, 20% Tween 80, 20% Triton X-100 or by mixture of these components (4 M urea, 1% Tween 80 and 1% Triton X-100), as described (Lavermicocca et al. 2000; Johnson 2013). The residual antifungal activity was measured by the method described above.

#### Reagents used and data processing

Agar–agar and yeast extract were from Becton, Dickinson and Co. (USA-France), peptone and MRS media were from Hi-Media (India). The other reagents used were from Sigma-Aldrich Co. (USA). Moreover, proteinase K from *Tritirachium album* with ≥ 30 units/mg, pepsin from porcine gastric mucosa with ≥ 250 units/mg and trypsin from bovine pancreas with ≥ 10,000 BAEE units/mg were from Sigma (USA).

All experiments were independently repeated three times. Mean values and standard deviations were calculated using Statgraphics software (Statpoint Technologies, Inc., Warrenton, VA, USA). Project for Statistical Computing version R 3.1.0 (The R Foundation of Statistical Computing, Vienna, Austria) was exploited to evaluate the statistical significance of obtained data by the Student’s t test, P < 0.05 if not mentioned.

## Results

### Screening of lactobacilli for antifungal activity

The primary screening of the antifungal activity among these strains has shown antifungal activity of lactobacilli strains MDC 9661, INA-5.1, INA-21.1, RIN-2003, MDC 9632 and MDC 9633 (Table 1). None of studied strains inhibited the growth of *G. candidum, T. viride* and *A. flavus.* The strains RIN-2003, MDC 9661, MDC 9632 and MDC 9633 demonstrated the antifungal activity against yeast *D. hansenii*.

**Table 1:** Antifungal activity of different LAB

Strain^a^	Fungi									
	*M. plumbeus*	*Fusarium* sp.	*Cladosporium* sp.	*G. candidum*	*A. flavus*	*P. aurantioviolaceum*	*T. viride*	*D. hansenii*	*C. guilliermondii*	*C. albicans*
BAM-2003-Lb	−^b^	−	−	−	−	−	−	−	+^b^	−
RIN-2003-Ls	−	+	+	−	−	−	−	+	+	−
MDC 9661	+	+	−	−	−	+	−	+	+	+
INRA-2010-H11	−	−	+	−	−	−	−	−	+	−
MDC 9632	−	+	+	−	−	−	−	+	−	−
INA-5.1	−	−	−	−	−	+	−	−	+	+
MDC 9633	−	+	+	−	−	−	−	+	−	−
INA-21.1	−	−	−	−	−	+	−	−	+	+

^a^For strains, see “Materials and methods”

^b^+ presence of antifungal activity, − absence of antifungal activity, ± elongation of spores generation

The inhibitory activity of *L. rhamnosus* MDC 9661 against *Penicillium aurantioviolaceum* and *Mucor plumbeus* is a new finding for LAB which might be of great significance.

**Fig. 1:**
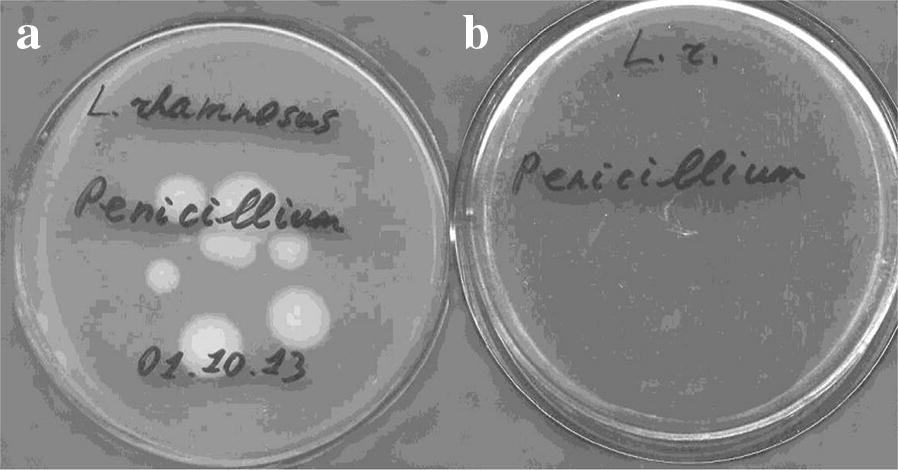
Antifungal activity of *L. rhamnosus* MDC 9661 strain. **A** after 24 h of cultivation, **B** after 48 h of cultivation. For details see “Materials and methods”

Interestingly, study of antifungal activity depended on LAB cultivation duration revealed that the overnight culture (stock culture) or culture which had been grown during 24 h showed no antifungal activity (Fig. 1). The antifungal activity was observed after 48 h of LAB growth (Fig. 1).

Thus, *L. rhamnosus* MDC 9661 isolated has a high inhibitory activity against different fungi growth; this strain was used in further study of such activity properties and its possible nature.

### Lactobacilli antifungal activity properties

The most important property of lactobacilli antifungal activity is its stability under different external factors. Indeed, MDC 9661 after 48 h of growth kept its activity against *P. aurantioviolaceum* and *M. plumbeus*in the wide range of pH from 3 to 10. But the treatment of MDC 9661’s cultural liquid after 48 h of growth at different temperature (of 45–80 °C) during 15 min revealed the high sensitivity of antifungal activity to temperature: MDC 9661 lost its activity against *P. aurantioviolaceum* after exposure to 60 °C and against *M. plumbeus*—after exposure to 45 °C.

**Fig. 2:**
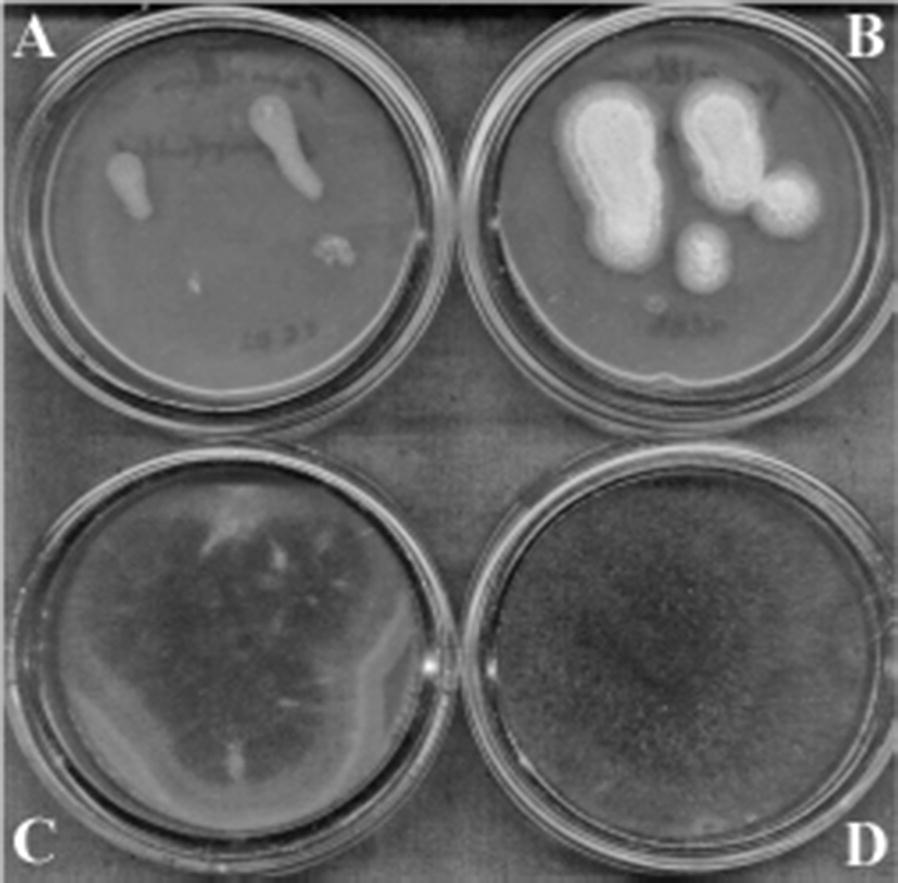
The antifungal activity of *L. rhamnosus* MDC 9661 cell-free cultural liquid. **A** MDC 9661 supernatant with 10^4^ spore/mL *P. aurantioviolaceum* spores, **B** control *P. aurantioviolaceum* without LAB supernatant, **C** MDC 9661 supernatant with 10^4^ spore/mL *M. plumbeus*, **D** control *M. plumbeus* without LAB supernatant. For details see “Materials and methods”

This research revealed that the cell free supernatant indicated no antifungal activity but the fungi growth was delayed (Fig. 2).

**Fig. 3:**
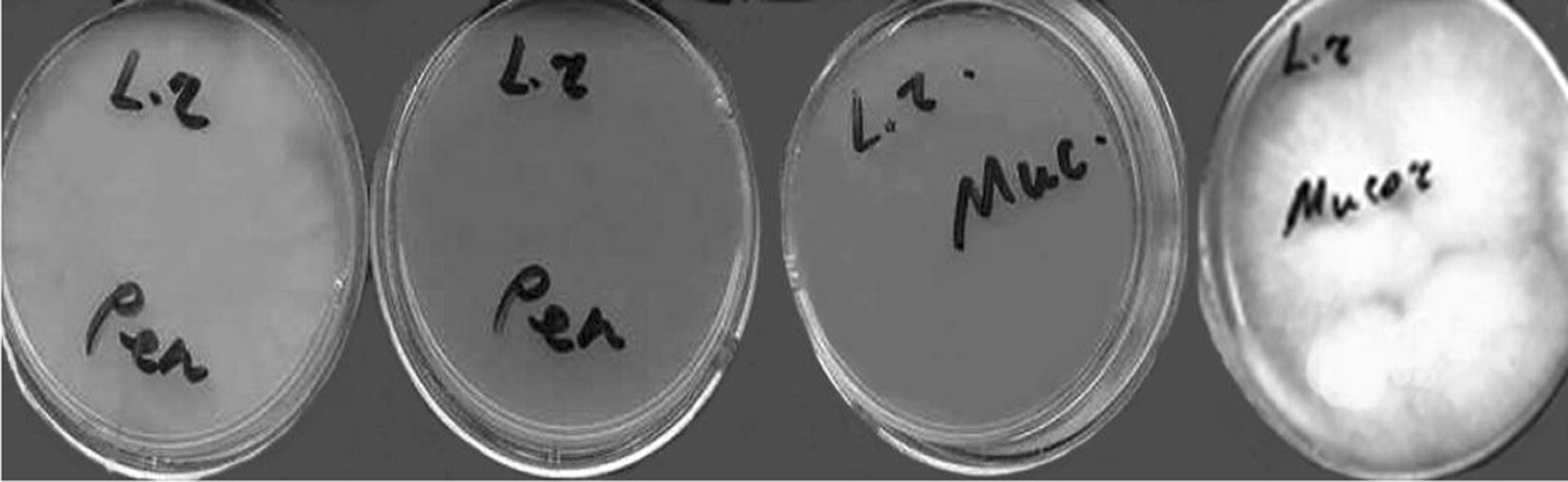
The antifungal activity of *L. rhamnosus* MDC 9661 disintegrated biomass. The supernatant of disintegrated biomass was added in Petri dishes where the growth of mycelium can be seen (the side) and the sediment of disintegrated biomass was added in Petri dishes where the growth of mycelium cannot be seen (in the middle of Fig.). For details see “Materials and methods”

Study of antifungal activity of adjusted to pH 6.5 supernatant concentrated (10×) by evaporation and concentrated (22.5×) by lyophilization did not show any positive results. The biomass kept its antifungal activity after treatment by both − 30 °C and lysozyme and after disintegration of cells by ultrasound (Fig. 3).

The auto-lysates of different fungi strains were used as elicitors. As it could be seen (see Fig. 3), all control samples showed good antifungal activity, but separation of biomass from supernatant led to the loss of activity.

### On the nature of lactobacilli antifungal activity

**Fig. 4:**
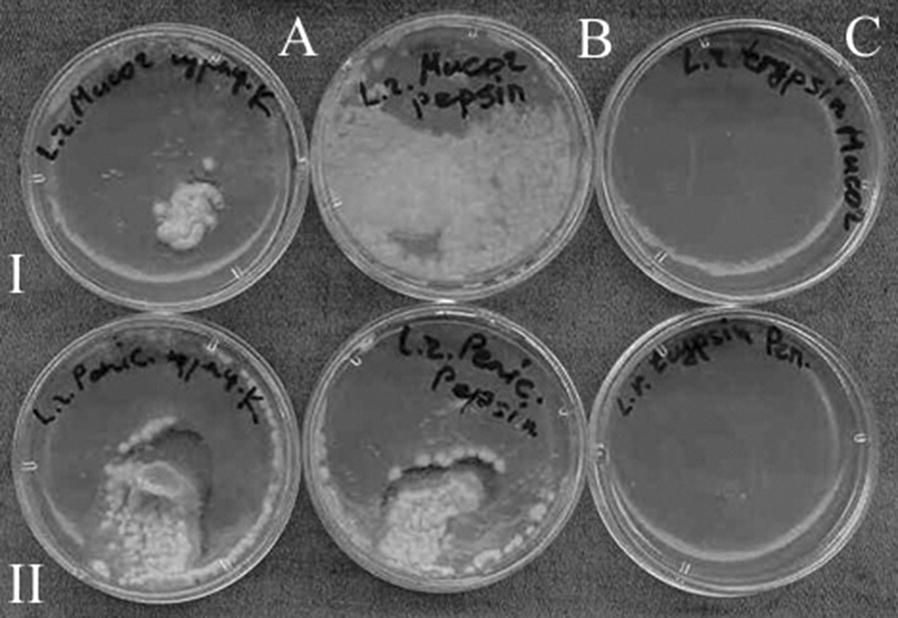
The effects of different enzymes on antifungal activity of *L. rhamnosus* MDC 9661. I line—inhibition of *M. plumbeus* growth. II line—inhibition of *P. aurantioviolaceum* growth. **A** MDC 9661 cultural liquid treated by proteinase K, **B** MDC 9661 cultural liquid treated by pepsin, **C** MDC 9661 cultural liquid treated by trypsin. For details see “Materials and methods”

The other part of the present work was devoted to the study of the nature of MDC 9661 antifungal activity. For this purpose, we used different enzymes, effects of which indicated the following findings (Fig. 4). The treatment of MDC 9661 culture by proteinase K and pepsin inhibited its antifungal activity against *M. plumbeus* and *P. aurantioviolaceum.* However, antifungal activity of MDC 9661 remained after the treatment with trypsin. These results allow to speculate on the proteinaceous nature of MDC 9661 antifungal activity probably had protein nature. However, protein nature of antifungal activity is interesting suggestion for lactobacilli; a further study is required.

## Discussion

Armenian dairy products are rich in proteins and important nutrients and can be a good source of unique combinations of LAB. The latter can produce several antimicrobials, proteolytic and antifungal compounds. The antibacterial and proteolytic activity of some isolated strains had been shown before (Bazukyan et al. 2010; Movsesyan et al. 2010). That is why it was interesting to study the antifungal activity as well. The investigation of antifungal activity showed the high specificity of LAB to inhibit the growth of fungi species. Like this, the strains RIN-2003, MDC 9661, MDC 9632 and MDC 9633 blocked the growth of *Fusarium* CB 1853, although the growth of *M. plumbeus* was inhibited by MDC 9661 strain only (see Table 1). Moreover, the growth of *P. aurantioviolaceum* and *C. albicans* was suppressed by MDC 9661, INA-5.1 and INA-21.1.

Determination of some properties of antifungal activity revealed the wide pH stability, opening great perspectives of its application in food industry. The pH stability of MDC 9661 antifungal activity differs from data reported by different groups. De Muynck et al. (2004) suggested that the adjustment of the supernatant of *L. acidophilus*, *L. amylovorus*, *L. brevis* and *L. coryniformis* subsp. *coryniformis* strains to pH values of 5.0, 5.5 and 6.0, indeed, inhibited the antifungal activity. In the study of Laref and Guessas (2013) the antifungal activity of *L. plantarum* strains remained on high level at pH 6.0 and pH 7.0, therefore the antifungal activity was suggested to be not only due to non-dissociated organic acids, but also by dissociated ones.

From the other side, the low temperature stability of antifungal activity of *L. rhamnosus* MDC 9661 limited its application. This property differed from the data reported by the other groups. Schwenninger et al. (2005) reported also that the heating of the supernatant of *Lactobacillus paracasei* subsp. *paracasei* for 10 min at 100 °C didn’t change the antifungal activity. Similar results were obtained by Gerez et al. (2009) of the *L. plantarum* supernatant. However, Gourama (1997) showed the loss of inhibitory activity at 100 °C for a treatment during 10 min. So, for application of the *L. rhamnosus* MDC 9661 antifungal activity in industry it should be used only after food thermic treatment.

The protein nature of lactobacilli antifungal activity was revealed by its treatment with different proteolytic enzymes. Interestingly, opposite results with antifungal activity of LAB were reported in some studies (Gerez et al. 2009; Lan et al. 2012; Niku-Paavola et al. 1999), where LAB treated with proteolytic enzymes (pepsine, pronase, proteinase K and α chymotrypsin) showed an inhibitory activity, suggesting that the antifungal activity could be not due to protein. However, Rouse et al. (2008) in their work with the concentrated supernatant of *L. plantarum*treated with proteinase K obtained essentially eliminated antifungal activity. The other authors (Guo et al. 2011; Mauch et al. 2010) demonstrated that the proteolytic treatment reduced antifungal activity of *L. brevis*and *L. reuteri*.

The finding that the treatment by trypsin did not reduce the antifungal activity of *L. rhamonus* MDC 9661 allows one to conclude the absence of lysine-arginine bands in the middle part of antifungal component (Rodriguez et al. 2008). This statement is correct only in the case of absence of proline link to either of these amino acids.

The preliminary purification of antifungal component synthesized by *L. rhamnosus* MDC 9661 revealed that neither 4 M urea, nor 20% Tween 80 and 20% Triton X-100 could separate the antifungal component from the cell wall components. This can be concluded after comparing the presence of fungi growth on the plates with the cell free supernatant after the treatment by ultrasound and 4 M urea, and the absence of fungi growth on the plates with the cell wall particles after the same treatment (Tables 2 and 3). The presence of both 20% Tween 80 and Triton X-100 in the media inhibited the growth of fungi; that is why the concentrations of these two components were decreased to 1%. The extraction of antifungal component from the cell wall particles was carried out by using the mixture of mentioned three components (see “Materials and methods”). But even this mixture could not separate the antifungal component (see Table 3). In order to fully understanding of the structure of the antifungal component and to study its properties, it is absolutely necessary to perform complete purification of component using special technique. Even in the case that the purification of antifungal component was not successful, MDC 9661 strain is of interest for current research and development.

**Table 2:** The residual antifungal activity of *L. rhamnosus* MDC 9661 after the treatment with ultrasound and 2 and 4 M urea

Sample	Treatment^a^	Antifungal activity against	
		*M. plumbeus*	*P. aurantioviolaceum*
Control, the media without LAB growth	–	+^b^	+
Not treated cell-free supernatant of MDC 9661	–	−^b^	−
Cell-free supernatant	Ultrasound	+	+
Cell wall particles of LAB	Ultrasound	−	−
Control media without LAB growth	2 M urea	+	+
Control media without LAB growth	4 M urea	+	+
Cell-free supernatant of MDC 9661	Ultrasound and 2 M urea	+	+
Cell wall particles of MDC 9661	Ultrasound and 2 M urea	−	−
Cell-free supernatant of MDC 9661	Ultrasound and 4 M urea	+	+
Cell wall particles of MDC 9661	Ultrasound and 4 M urea	−	−

^a^See “Materials and methods”

^b^+ is the presence of mold’s growth, − is mold’s growth inhibition

**Table 3:** The residual antifungal activity of *L. rhamnosus* MDC 9661 after the treatment with 1% Tween 80 and 1% Triton X-100

Sample	Treatment^a^	Antifungal activity against	
		*M. plumbeus*	*P. aurantioviolaceum*
Control, the media without LAB growth	–	+^b^	+
Control, the media without LAB growth	1% of Triton X-100	+	+
Control media without LAB growth	1% of Tween 80	+	+
Cell wall particles of LAB	Ultrasound	+	−^b^
Cell-free supernatant	Ultrasound	+	+
Cell wall particles of MDC 9661	Ultrasound and 1% Triton X-100	−	−
Cell-free supernatant of MDC 9661	Ultrasound and 1% Triton X-100	+	+
Cell wall particles of MDC 9661	Ultrasound and 1% Tween 80	+	+
Cell-free supernatant of MDC 9661	Ultrasound and 1% Tween 80	+	+

^a^See “Materials and methods”

^b^+ is the presence of mold’s growth, − is mold’s growth inhibition

Based on the results obtained, it can be concluded that lactobacilli strains isolated from Armenian dairy products demonstrated inhibitory activity against different fungi, especially *P. aurantioviolaceum* and *M. plumbeus*. *L. rhamnosus* MDC 9661 showed the maximum antifungal activity when cultivated in modified MRS during 48 h. This activity was stable to different pH values and sensitive to high temperature but could be lowered by proteolytic enzymes. The activity links with bacterial cell wall and has a protein nature; a further purification of which is required. MDC 9661 can be applied as starter for production of dairy products, functional food and preserving strain in food production.

## Abbreviations

LAB: lactic acid bacteria
FFIRL: Biopolymers Interaction Assemble, Function and Interaction of Proteins Laboratory
INRA: National Research Institute of Agronomy
MDC: Microbial Depository Center
MRS: De Man, Rogosa and Sharpe

## References

[CR1] Ahmadova A, Dimitrov-Todorov S, Hadji-Sfaxi I, Choiset Y, Rabesona H, Messaoudi S, Kuliyev A, de Melo Gombossy, Franco BD, Chobert J-M, Haertlé T (2013). Antimicrobial and antifungal activities of *Lactobacillus curvatus* strain isolated from homemade Azerbaijani cheese. Anaerobe.

[CR2] Bazukyan I (2018). Identification and comparative characterization of new lactic acid bacteria isolated from Armenian dairy products by phenotypic and molecular methods. Proc Yerevan State Univ Chem Biol.

[CR3] Bazukyan I, Ahabekyan N, Madoyan R, Dalgalarrondo M, Chobert JM, ChoPopovbert Yu, Haertlé T, Mendez-Vilas A (2010). Study of cell envelope proteinase systems of natural isolated thermophilic lactobacilli. Proceedings of BioMicroWorld conference on Microorganisms in Industry and Environment: from scientific and industrial research to consumer products.

[CR4] Dalie DKD, Deschamps AM, Richard-Forget F (2010). Lactic acid bacteria-potential for control of mould growth and mycotoxins. Food Control.

[CR5] De Muynck C, Leroy AIJ, De Maeseneire S, Arnaut F, Soetaert W, Vandamme EJ (2004). Potential of selected lactic acid bacteria to produce food compatible antifungal metabolites. Microbiol Res.

[CR6] Falguni P, Shilpa V, Mann B (2010). Production of proteinaceous antifungal substances from *Lactobacillus brevis* NCDC. Int J Dairy Technol.

[CR7] Gerez CL, Torino MI, Rollan G, De Valdez GF (2009). Prevention of bread mould spoilage by using lactic acid bacteria with antifungal properties. Food Control.

[CR8] Gourama H (1997). Inhibition of growth and mycotoxin production of *Penicillium*by *Lactobacillus* species. Food Sci Techn.

[CR9] Guo J, Mauch A, Galle S, Murphy P, Arendt EK, Coffey A (2011). Inhibition of growth of *Trichophyton tonsurans* by *Lactobacillus reuteri*. J App Microb.

[CR10] Johnson M (2013). Detergents: Triton X-100, Tween-20, and More. Mater Methods.

[CR11] Lan W, Chen Y, Hui-chung W, Yanagida F (2012). Bio-protective potential of lactic acid bacteria isolated from fermented wax gourd. Folia Microbiol.

[CR12] Laref N, Guessas B (2013). Antifungal activity of newly isolates of lactic acid bacteria. Innov Rom Food Biotechnol.

[CR13] Lavermicocca P, Valerio F, Evidente A, Lazzaroni S, Corsetti A, Gobbetti M (2000). Purification and characterization of novel antifungal compounds from the sourdough *Lactobacillus plantarum* strain 21B. App Environ Microbiol.

[CR14] Mauch A, Dal Bello F, Coffey A, Arendt EK (2010). The use of *Lactobacillus brevis* PS1 to in vitro inhibit the outgrowth of *Fusarium culmorum* and other common *Fusarium* species found on barley. Int J Food Microbiol.

[CR15] Movsesyan I, Ahabekyan N, Bazukyan I, Madoyan R, Dalgalarrondo M, Chobert JM, Popov Yu, Haertlé T (2010). Properties and survival under simulated gastrointestinal conditions of lactic acid bacteria isolated from Armenian cheeses and matsuns. Biotech Biotechnol Equip.

[CR16] Navasardyan L, Marutyan S, Hovnanyan K, Trchounian A (2017). Survival and changes in morphology, mitotic and metabolic activity of yeast *Candida guilliermondii* exposed to X-irradiation. Ind J Biochem Biophys.

[CR17] Ndagano D, Lamoureux T, Dortu C, Vandermoten S, Thonart P (2011). Antifungal activity of 2 lactic acid bacteria of the *Weissella* genus isolated from food. J Food Sci.

[CR18] Niku-Paavola ML, Laitila A, Mattila-Sandholm T, Haikara A (1999). New types of antimicrobial compounds produced by *Lactobacillus plantarum*. J Appl Microbiol.

[CR19] Rodriguez J, Gupta N, Smith RD, Pevzner PA (2008). Does trypsin cut before proline?. J Proteome Res.

[CR20] Rouse S, Harnett D, Vaughan A, Van Sinderen D (2008). Lactic acid bacteria with potential to eliminate fungal spoilage in foods. J Appl Microbiol.

[CR21] Schwenninger SM, Ah UV, Niederer B, Teuber M, Meile L (2005). Detection of antifungal properties in *Lactobacillus paracasei* subsp. *paracasei* SM 20, SM 29, and SM 63 and molecular typing of the strains. J Food Prot.

